# Management of Shallow Vestibule with Reduced Attached Gingiva in Fixed Prosthetic Intervention

**DOI:** 10.7759/cureus.4975

**Published:** 2019-06-23

**Authors:** Shanmuganathan Natarajan, Fathima Banu, Madhan Kumar, Vamsi Lavu

**Affiliations:** 1 Prosthodontics, Faculty of Dental Sciences, Sri Ramachandra University, Chennai, IND; 2 Periodontics, Faculty of Dental Sciences, Sri Ramachandra University, Chennai, IND

**Keywords:** vestibuloplasty, fixed prosthesis, lefort fracture

## Abstract

Shallow vestibule has long been considered a deterring factor in the use of removable dental prosthetics. The need for management in fixed prosthetic replacement is not widely discussed. Adequate attached gingiva is essential for continued proper oral hygiene. Muscular and fibrous traction leads to gingival recession, which can cause marginal leakage in a fixed prosthetic restoration. In the long-term, this causes the suprastructure to fail and, ultimately, the restoration also fails. Therefore, shallow vestibule with reduced attached gingiva should be identified in the diagnostic stage and should be effectively managed prior to restoration of lost tooth structure. This case report discusses the management of a shallow vestibule in a LeFort I fracture with emphasis on a fixed prosthetic replacement.

## Introduction

Tooth, gingiva, and periodontal ligament form a composite unit. The overall goal of prosthodontic replacement encompasses both functional and aesthetic appearance along with prosthesis longevity. While shallow vestibule does not deter fixed prosthetic replacement, it causes food impaction against the gingival margin and into the interproximal spaces, leading to poor plaque control [1]. Several studies indicate that an adequate width of attached gingiva is essential for the continuation of proper oral hygiene. Wennstrom and Piniprato stated that a combination of the shallow vestibule and insufficient width of attached gingiva might favor the accumulation of food during mastication and presents a barrier to maintaining good oral hygiene [2]. Muscular and fibrous attachment causes gingival traction due to inadequate attached gingiva and shallow vestibule, further advancing gingival recession and plaque accumulation [3]. Moreover, increasing the attached gingiva would reduce inflammation around the restored teeth, and gingival margin binds better around teeth and implants with attached gingiva [4]. This case report explains the management of a partially edentulous patient with reduced attached gingiva.

## Case presentation

**Figure 1:**
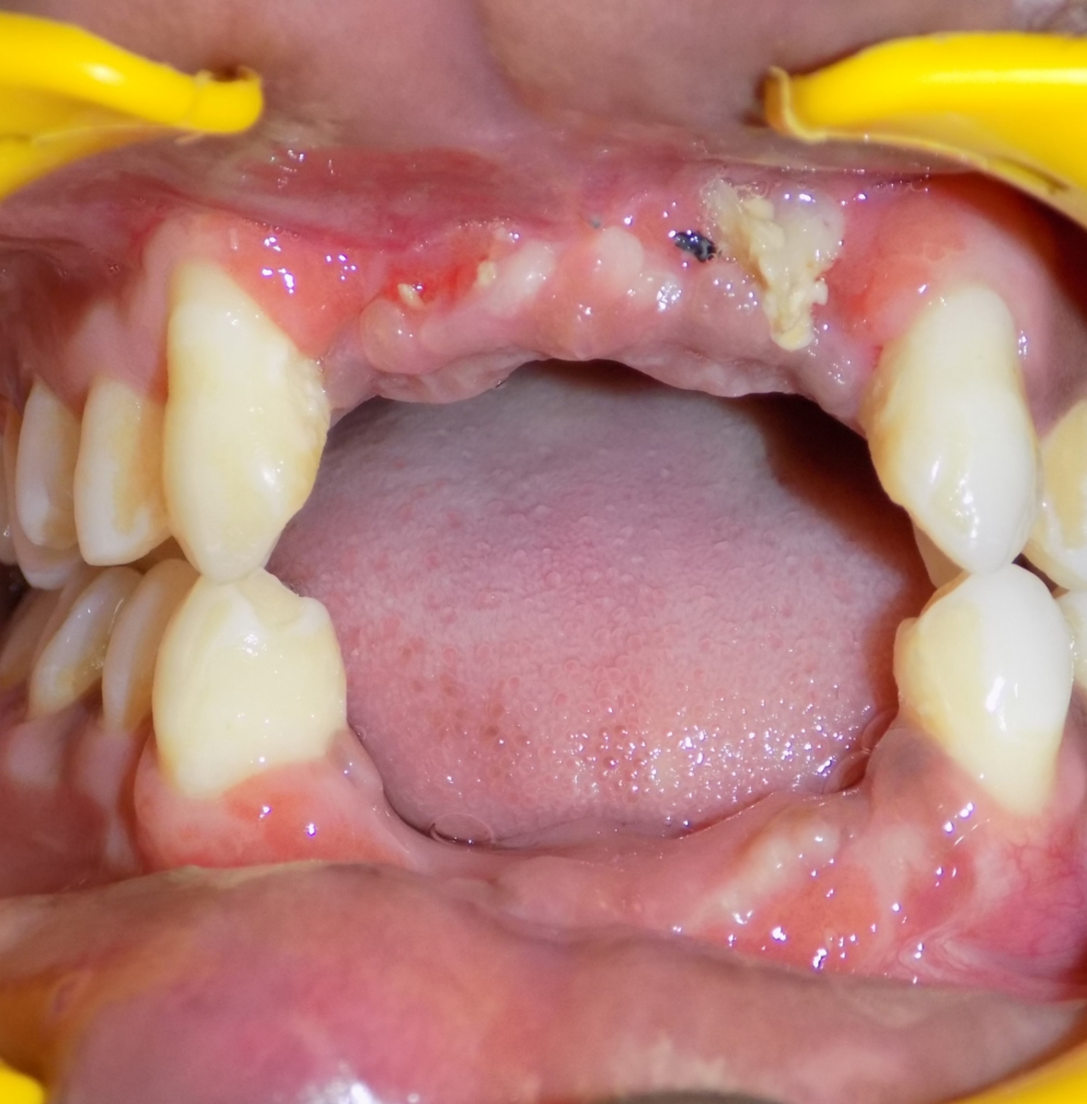
Post-surgical intraoral view

A 19-year-old male patient presented to the Department of Oral Maxillofacial Surgery following a road traffic injury. He had a LeFort I fracture with avulsed 11,12,21,22 and fracture of the left parasymphysis mandible with avulsed 31,32,41,42. Open reduction and internal fixation of the LeFort I and left parasymphysis fracture was done under general anesthesia. One month after the procedure, the patient sought replacement of the missing anterior teeth (Figure 1).

**Figure 2:**
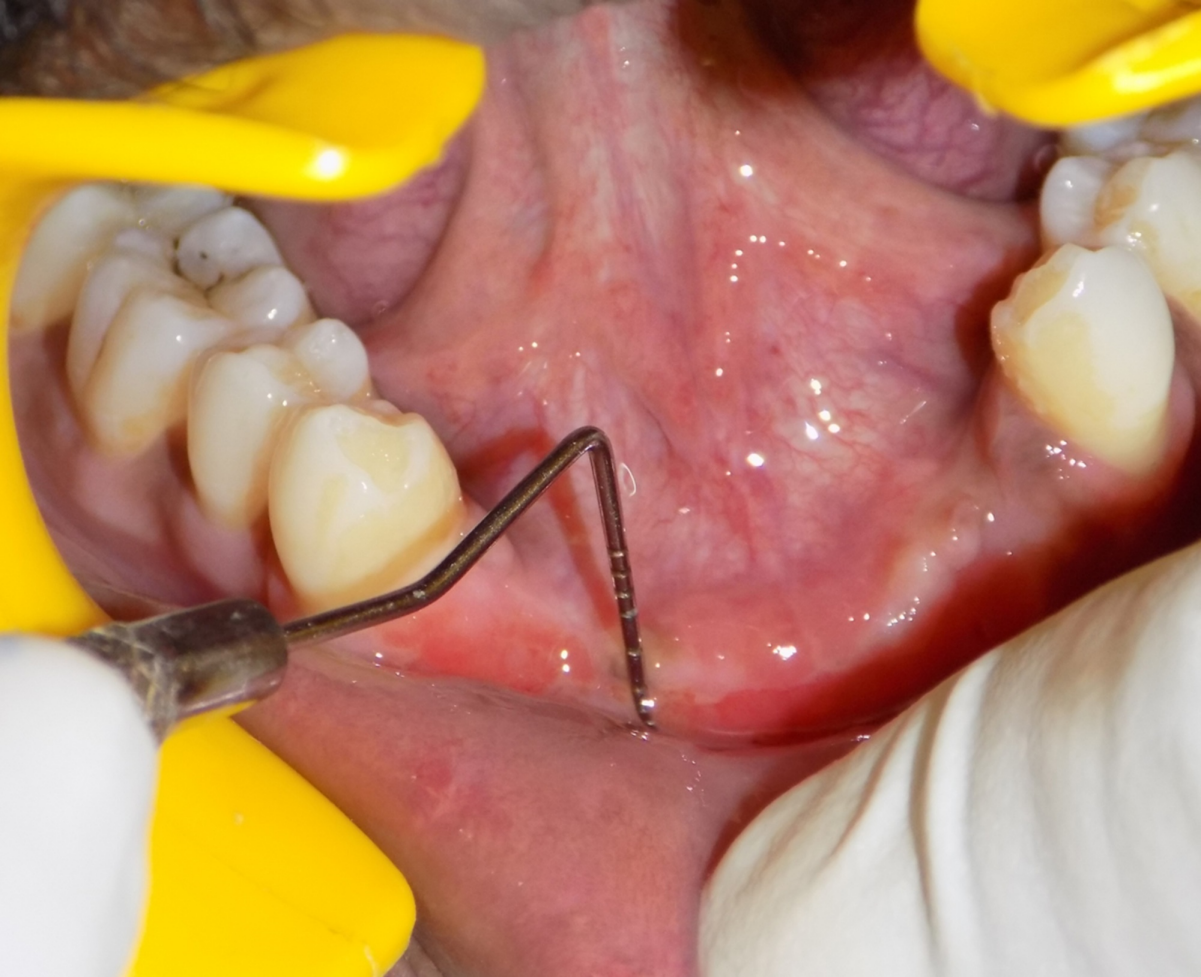
Reduced vestibular depth

On clinical evaluation, we noted reduced vestibular depth and attached gingiva in both the maxilla and mandible (Figure 2).

**Figure 3:**
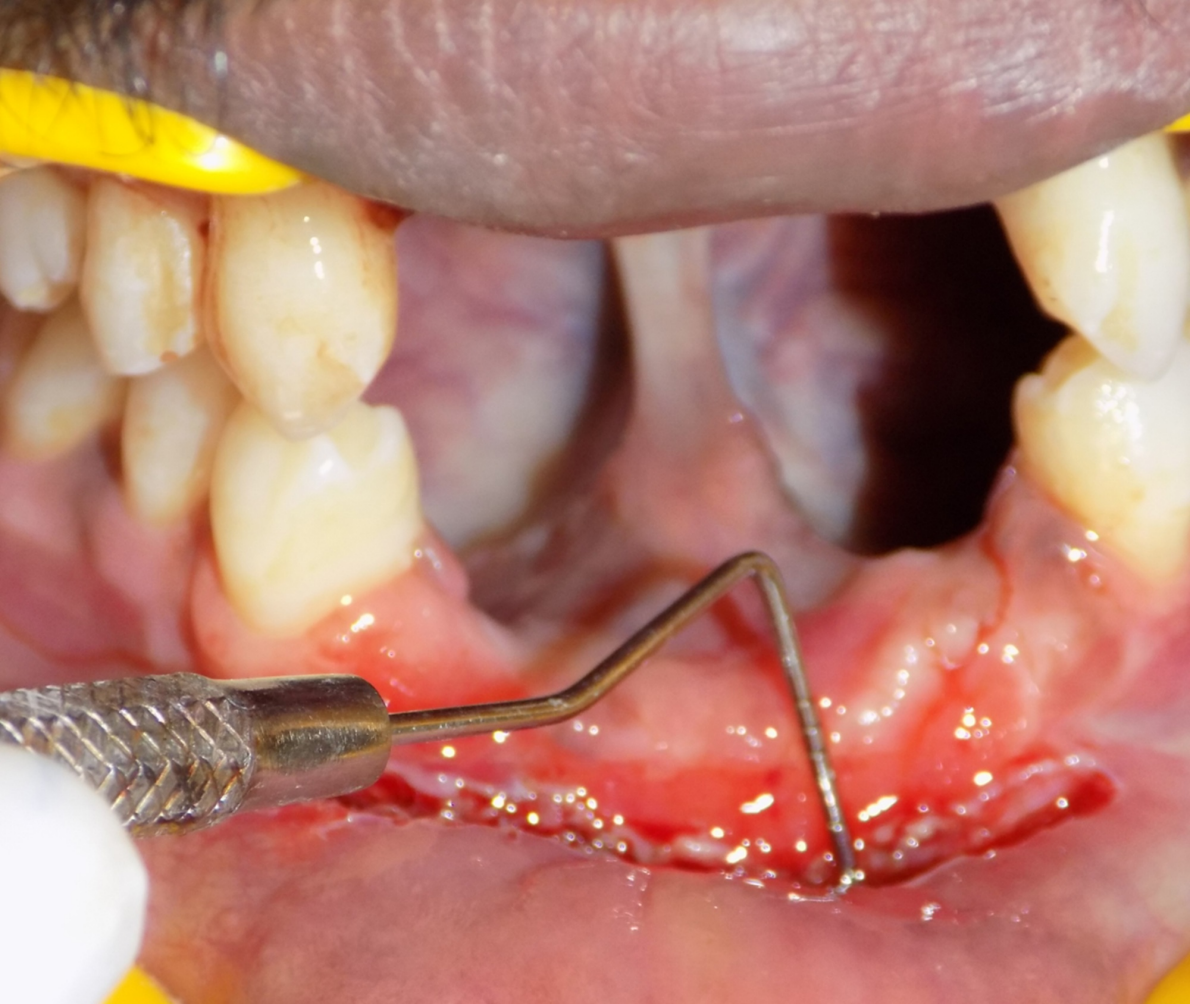
Vestibuloplasty via electrosurgery

To maintain aesthetics and improve the prognosis of the prosthesis, vestibuloplasty was planned followed by fixed partial denture for the edentulous maxillary and mandibular anterior teeth. Using the diagnostic cast, a temporary removable prosthesis was fabricated after which vestibuloplasty was performed. Vestibular deepening was performed via electrosurgery. A combination of infra-orbital nerve blocks and nasopalatine nerve blocks was administered with 2% lidocaine (1:200,000 adrenaline). The periosteal fenestration method was used for vestibular deepening. A needle electrode performed a combination of cutting and coagulation currents and supraperiosteal dissection of the muscle attachments in the labial vestibular area from 13-23 and 33-43. Once a depth of 7 mm was achieved, a horizontal incision was made in the periosteum, ensuring minimal contact with the alveolar bone to prevent necrosis (Figure 3).

**Figure 4:**
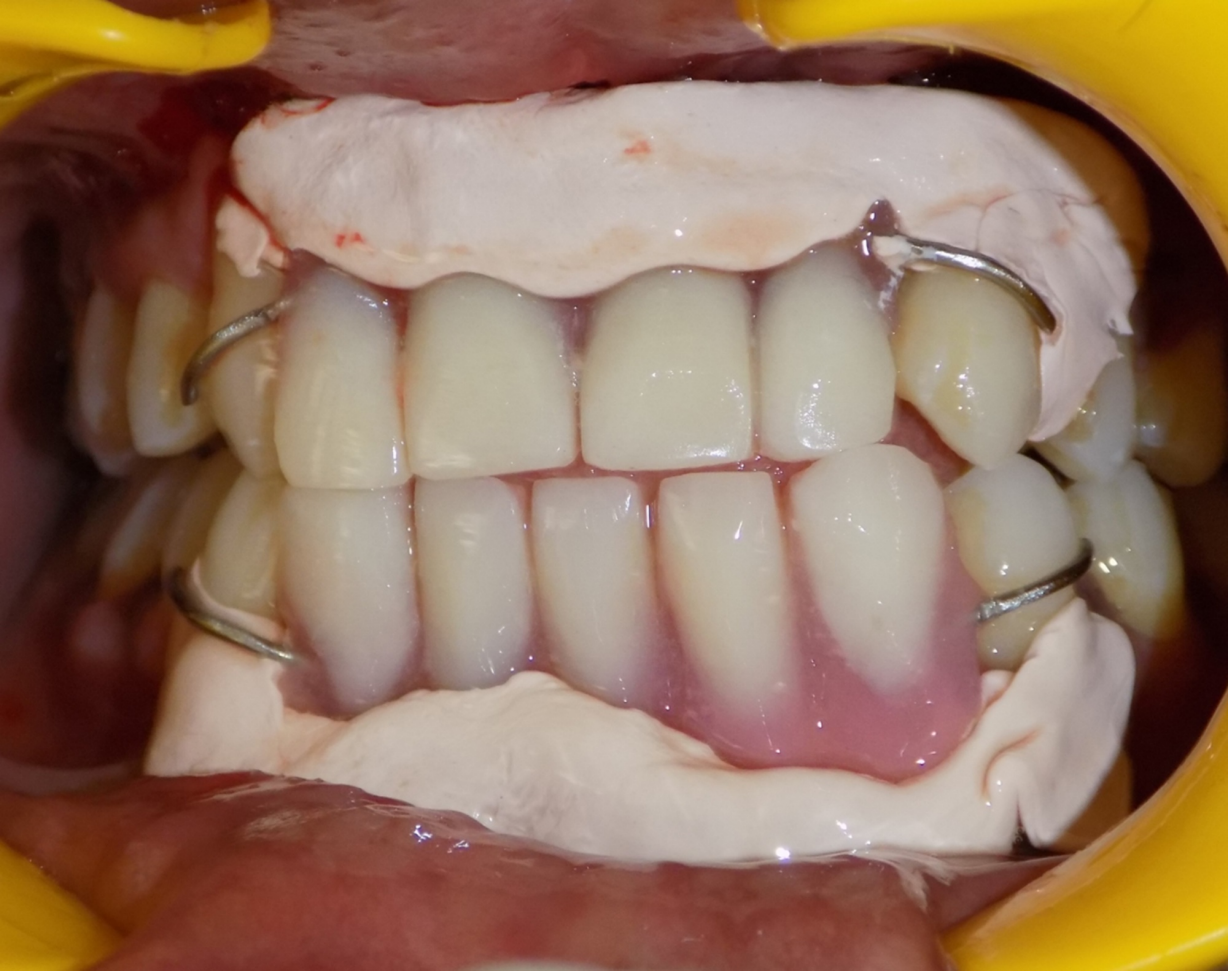
Temporary partial denture with Perio Pack

Irrigation was done with sterile saline, and a periodontal dressing (Coe Pak™ Automix; GC America, Inc., Alsip, IL) was placed. After three weeks, the healing was deemed satisfactory for further final prosthetic work. The temporary removable prosthesis was used as a postsurgical splint to prevent relapse until the healing phase (Figure 4).

**Figure 5:**
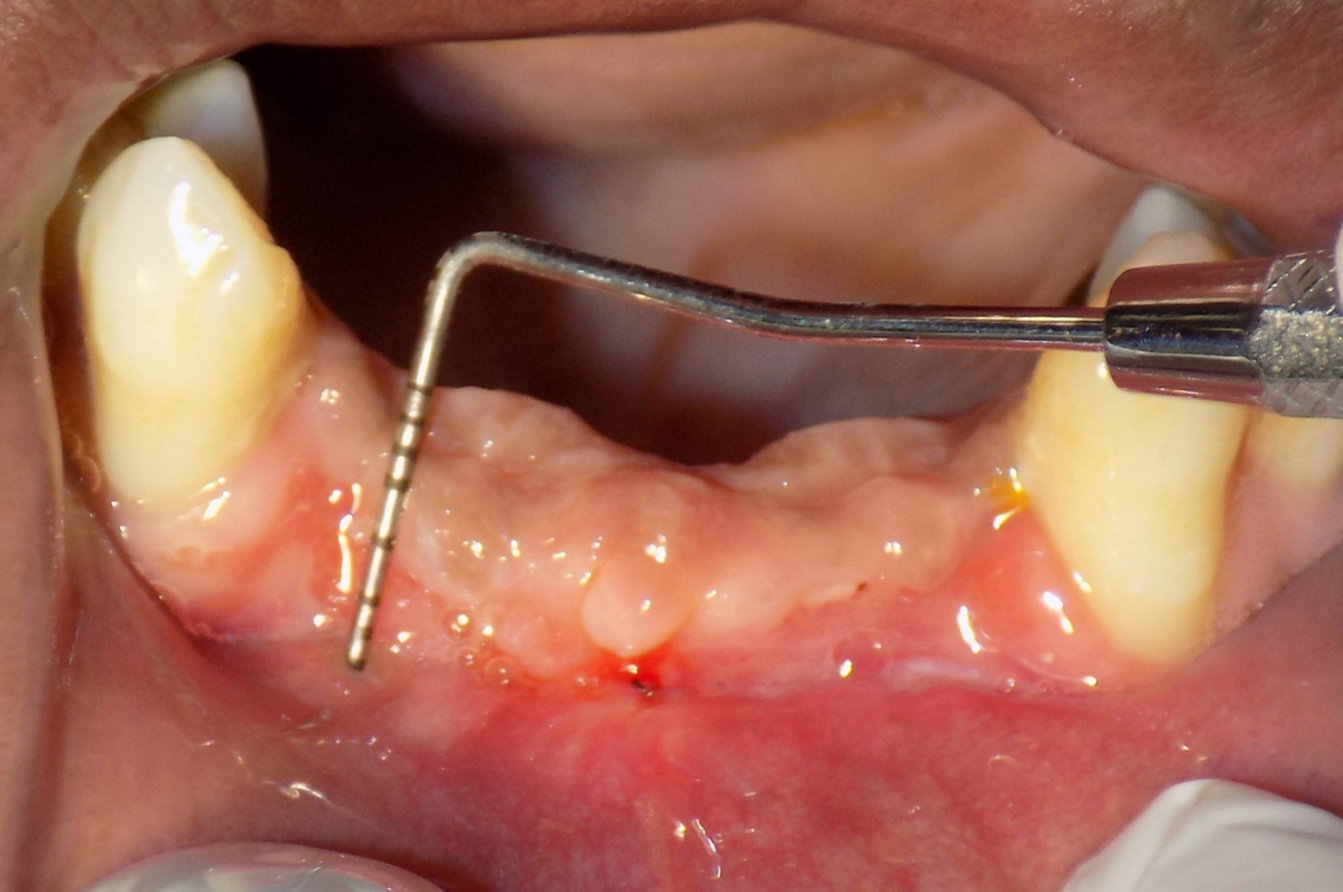
Post-vestibuloplasty in maxillary arch

**Figure 6:**
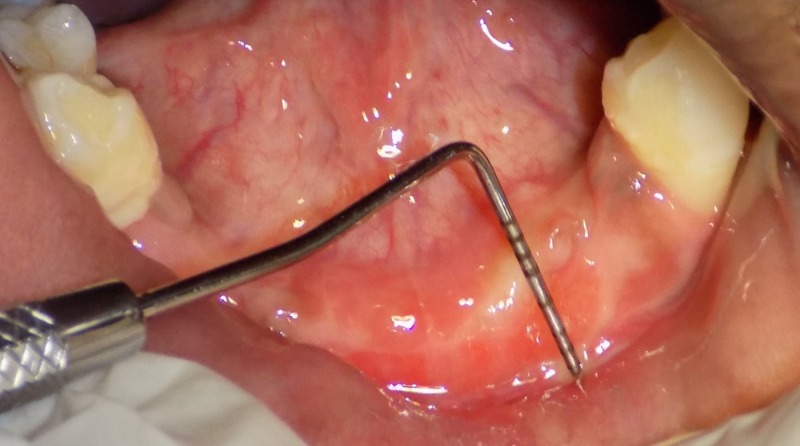
Post-vestibuloplasty in mandibular arch

After four weeks, the patient was recalled for evaluation of the surgery (Figures 5, 6).

**Figure 7:**
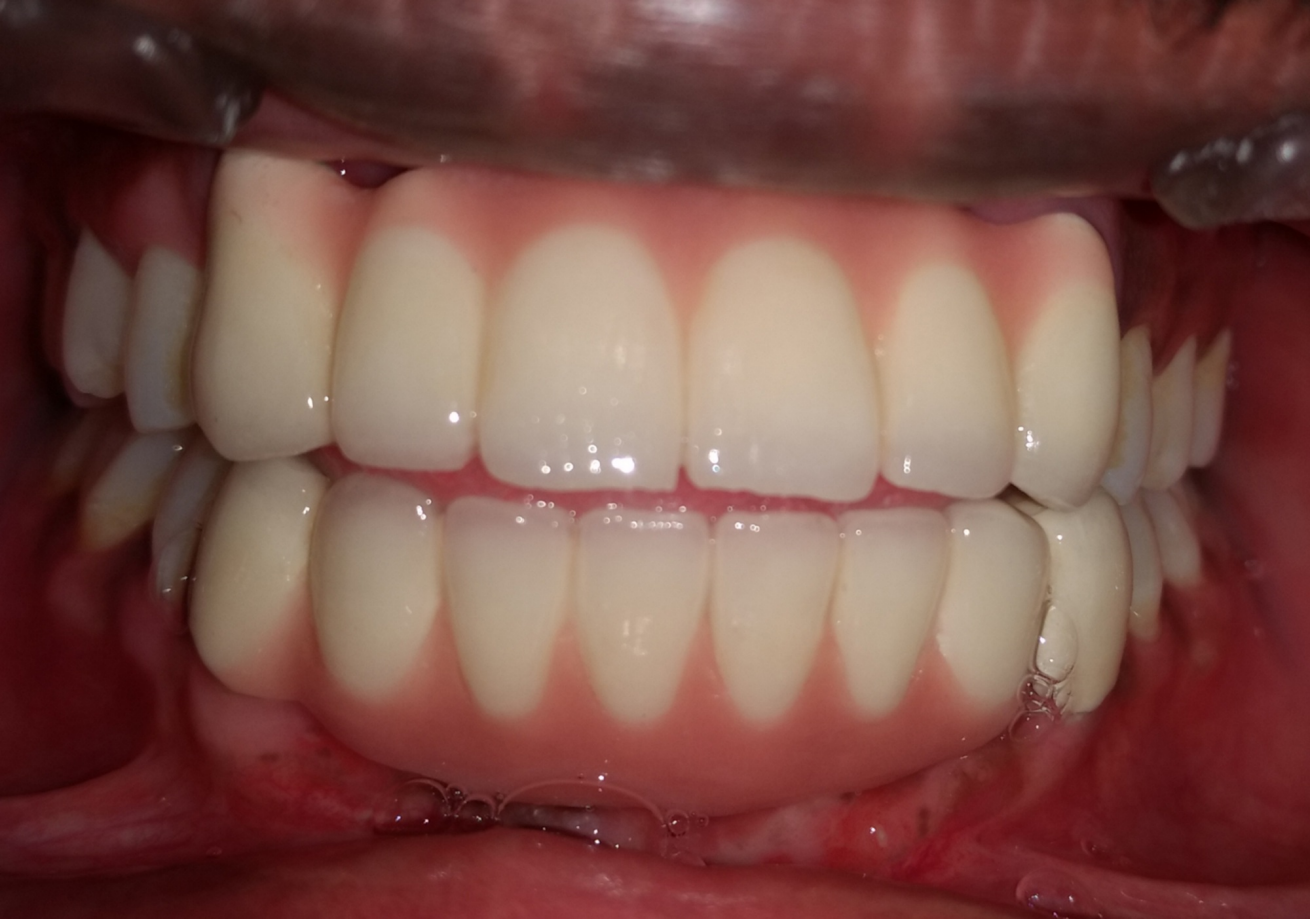
Review of fixed partial denture at one year postoperatively

A relapse of a few millimeters is expected due to tissue contraction during healing. Preparation of abutment teeth 13,23, 33,43 for fixed partial denture was done and an impression made. Final cementation of the metal-ceramic fixed partial denture was luted with zinc polycarboxylate cement. After a year of final cementation, review of the final prosthesis and the associated structures showed good maintenance (Figure 7).

## Discussion

The prognosis of a fixed partial denture is poor due to marginal leakage, unaesthetic appearance, and dentinal hypersensitivity given the higher degree of susceptibility associated with gingival recession [3]. In such a situation, vestibuloplasty is mandatory for cases requiring fixed prosthesis. The prognosis of submucosal vestibuloplasty is subjected to a satisfactory level of bone and mobile mucosa so that surgical extension of the vestibule can be attained without tautness [5,6]. In the present case report, the patient had lost the vestibular depth due to the LeFort I fracture and subsequent repositioning. Hence, the electrosurgery method of vestibuloplasty was done to enable vestibular deepening along with increasing the attached gingiva. In 1969, Wade reported that adequate width of attached gingiva is a common requirement for root coverage. Hence, vestibular deepening is an effective technique for gaining the width of the attached gingiva and avoiding gingival recession [7].

Several techniques have been developed since 1956, but scar formation and relapse are the most frequent complications in vestibular deepening due to exposure of the bone [8]. The periosteal fenestration method prevented necrosis of the site. Because the muscle attachment and overlying tissue are fenestrated, the relapse of vestibular deepening was negligible. Hence, the prognosis of the fixed prosthesis is good. Until now, vestibular deepening was considered important in considering removable prosthesis wherein adequate flange extension was found to be necessary for retention and stability of the prosthesis. Little interest exists for fixed prosthesis cases with shallow vestibule, and therefore, failure due to gingival recession and root caries persists. This case report emphasizes the importance of vestibular deepening in fixed prosthesis cases with shallow vestibule and reduced attached gingiva.

## Conclusions

The importance of maintaining adequate attached gingiva has often been overlooked during treatment planning with fixed prosthesis cases. Prolonged muscle traction, especially in cases with reduced attached gingiva, causes gingival recession. Until now, the management of shallow vestibule has largely been considered important only when replacing it with a removable prosthetic system for retentive purposes. However, failure to identify the effect of shallow vestibule leads to development of marginal gap, sensitivity, and caries in the abutment teeth. To prevent scar formation, deepening of the vestibule was done by the periosteal fenestration method which led to the success of the fixed prosthesis. Dental surgeons must treat the defect before prosthetic intervention to ensure prosthetic success in the long-term.
